# Dr. Ida C. Bender

**Published:** 1916-07

**Authors:** 


**Dr. Ida C. Bender,** Buffalo 1890, died at her home in Buffalo, June 11. She was the eldest child of the late Philip Henry Bender, proprietor of the Buffalo Telegraph, which was later merged with the Freie Presse. She was graduated with the highest honors at the Buffalo Normal School, winning the Jesse Ketchum gold medal. Her life work was that of a teacher, of languages at the Normal, in grade work in the public schools, and as Principal of the Normal School of Practice. She was one of quite a number of Buffalo teachers who, about 1890, entered the medical department of the University of Buffalo, some afterward engaging in practice, others taking the course for its educational value and to be able to add to their value as teachers, a thorough knowledge of hygiene and the ability to detect physical abnormalities in pupils, and to give advice as to the removal of these educational handicaps. This was, indeed, at least locally, the beginning of the system of medical inspection of school children. During her course of study in medicine, Miss Bender assumed the teaching of physiology and other scientific branches in the Buffalo Central (now Hutchinson) High School, succeeding the late Charles Linden. Shortly after her graduation with honors at the University of Buffalo, Dr. Bender was appointed Supervisor of the Primary Grades of the Buffalo Public Schools, a position created at the time of the reconstruction and modernization of the department by the Superintendent, Henry P. Emerson. In this work, Dr. Bender continued for a little over a quarter of a century, until her death, although she had been incapacitated for nearly a year. She was largely instrumental in the elevation of the profession of teaching, in many ways, by organization, insistance on post graduate study of theoretic and practical kinds along lines well developed by the medical profession, by forwarding the plan of pensions and in many other ways. Although thus nominally an executive officer, Dr. Bender always remained a teacher. Many who were in school after her promotion from class work, remember her from her visits to the schools in the capacity of Supervisor and recall the actual information and insight into learning which they received from these brief periods of instruction. Intellectually and socially, she was a woman of the highest gifts, was widely known and every casual acquaintance became her friend.

